# Ocean Warming, More than Acidification, Reduces Shell Strength in a Commercial Shellfish Species during Food Limitation

**DOI:** 10.1371/journal.pone.0086764

**Published:** 2014-01-28

**Authors:** Clara L. Mackenzie, Graham A. Ormondroyd, Simon F. Curling, Richard J. Ball, Nia M. Whiteley, Shelagh K. Malham

**Affiliations:** 1 School of Ocean Sciences, College of Natural Sciences, Bangor University, Menai Bridge, Anglesey, United Kingdom; 2 Biocomposites Centre, Bangor University, Bangor, Gwynedd, United Kingdom; 3 Department of Architecture and Civil Engineering, University of Bath, Bath, Somerset, United Kingdom; 4 School of Biological Sciences, College of Natural Sciences, Bangor University, Bangor, Gwynedd, United Kingdom; Institute of Marine Research, Norway

## Abstract

Ocean surface pH levels are predicted to fall by 0.3–0.4 pH units by the end of the century and are likely to coincide with an increase in sea surface temperature of 2–4°C. The combined effect of ocean acidification and warming on the functional properties of bivalve shells is largely unknown and of growing concern as the shell provides protection from mechanical and environmental challenges. We examined the effects of near-future pH (ambient pH –0.4 pH units) and warming (ambient temperature +4°C) on the shells of the commercially important bivalve, *Mytilus edulis* when fed for a limited period (4–6 h day^−1^). After six months exposure, warming, but not acidification, significantly reduced shell strength determined as reductions in the maximum load endured by the shells. However, acidification resulted in a reduction in shell flex before failure. Reductions in shell strength with warming could not be explained by alterations in morphology, or shell composition but were accompanied by reductions in shell surface area, and by a fall in whole-body condition index. It appears that warming has an indirect effect on shell strength by re-allocating energy from shell formation to support temperature-related increases in maintenance costs, especially as food supply was limited and the mussels were probably relying on internal energy reserves. The maintenance of shell strength despite seawater acidification suggests that biomineralisation processes are unaffected by the associated changes in CaCO_3_ saturation levels. We conclude that under near-future climate change conditions, ocean warming will pose a greater risk to shell integrity in *M. edulis* than ocean acidification when food availability is limited.

## Introduction

Ocean acidification (OA) has been reported to affect calcification processes in the shells of bivalve species by decreasing rates of calcium carbonate precipitation and/or increasing dissolution [1], [2], [3]. At the levels of pH reduction predicted for the end of the century, there are also many reports of the maintenance of calcification rates and shell growth despite reductions in pH and associated changes in CaCO_3_ saturation levels, especially in the blue mussel, *Mytilus edulis* (reviewed by Gazeau *et al.* [4]). Maintenance of shell growth, however, may compromise shell function as compensation for increases in shell dissolution under OA conditions can be energetically costly and compromise other homeostatic functions [5] with more extensive corrosion of the shell when food supply is limited [2]. The resulting effects of OA on mechanical strength in bivalve shells is poorly understood with most studies focusing on early life stages and the development of shell formation [6], [7], [8]. These studies suggest that short to medium term exposure to decreases in pH of >/ = 0.4 pH units can thin and weaken the bivalve shell [9], [10], [11]. As OA is not occurring in isolation from other environmental factors, there is an increasing realisation that concomitant changes in the environment are much more relevant to the conditions experienced by marine calcifiers in their natural environment, such as those predicted to occur as a result of climate change [12]–[15]. For example, a decrease in ocean pH by 0.2 to 0.4 pH units by the end of the century under the IPCC IS92a CO_2_ emission scenario [16], [17] is likely to coincide with an increase in mean sea surface temperature of 2–4°C [16], [18].

Temperature is a key environmental variable and has a significant effect on biomineralization processes and growth in bivalve shells (reviewed by Gazeau *et al.* [4]). Generally, temperature has a positive linear effect on shell growth [19], and can also result in changes in mineral composition. For example, shells built of the aragonite polymorph of calcium carbonate are more susceptible to dissolution at low temperatures [20], which may lead to a thinning of the shells [21], and could explain why shells in cold-water bivalves are characterised by an increase in the whole-shell calcite:aragonite ratio due to an increase in calcite, the less soluble but mechanically weaker polymorph of calcium carbonate [22]. More recently it has also been reported that temperature may also have an effect on shell microstructure by altering tablet width, thickness and angle spread of aragonite crystals in the nacreous structure of molluscan shells [23]. The functional significance of the observed changes, however, remains unclear [23]. In addition, the combined effects of OA and warming on the mechanical properties of the shells in adult bivalve molluscs over an extended exposure period (i.e. months) are largely unknown. Limited research on other taxa suggests that an elevation in temperature during OA is likely to have a negative synergistic effect on calcification processes with reductions in pH increasing the sensitivity of some species [13], [14], [24].

The effects of ocean warming and acidification on commercial shellfish species are of growing concern as molluscs and crustaceans comprise the majority of global seafood production [25]. Species such as the blue mussel *Mytilus edulis* represent an important regional and global resource with worldwide mussel fisheries estimated to be worth 390 million ($US) [26]. *M. edulis* also provides a number of important ecosystem services including uptake and recycling of energy and nutrients (benthic-pelagic coupling), bioturbation and bioirrigation of marine sediments, sediment/shoreline stabilization and habitat formation [27], [28]. The survival and health of *M. edulis* is dependent on its shell which performs a range of functions from providing primary defence against predators to delivering protection from physical environmental stressors. It also acts as a skeleton for muscle attachments and prevents sediment from collecting in the mantle cavity [29]. Additionally, the bivalve shell plays an important role in physiological homeostasis [30], [31]. Therefore any reduction in shell thickness, mechanical strength or alterations in shell microstructure and composition could have a profound effect on survival, not only by reducing protection of the soft tissues from predators and anthropogenic activity, but by also influencing the ability of *M. edulis* to respond to environmental change.

The purpose of the current study was to examine the combined effects of near-future OA and warming on the morphology, shell composition and surface area of adult *M. edulis* shells, as well as to examine the resulting effects on their functional properties by determining mechanical strength and flexibility. Mussels were collected from a sub-tidal population not normally exposed to extreme pH fluctuations, and exposed to an elevation in seawater temperature (warming) and reduction in pH (acidification) to match the levels predicted for 2100, either alone or in combination. Measurements were taken after six months exposure to avoid the most immediate effects of environmental change and to assess the ability of the mussels to compensate and maintain the functional properties of their shells in the longer-term. Mussels were fed 4–6 h day^−1^ in order to assess the functional repercussions of limited food availability which is relevant for future climate change scenarios where food is in short supply.

## Materials and Methods

### Animal Collection

**Figure 1:**
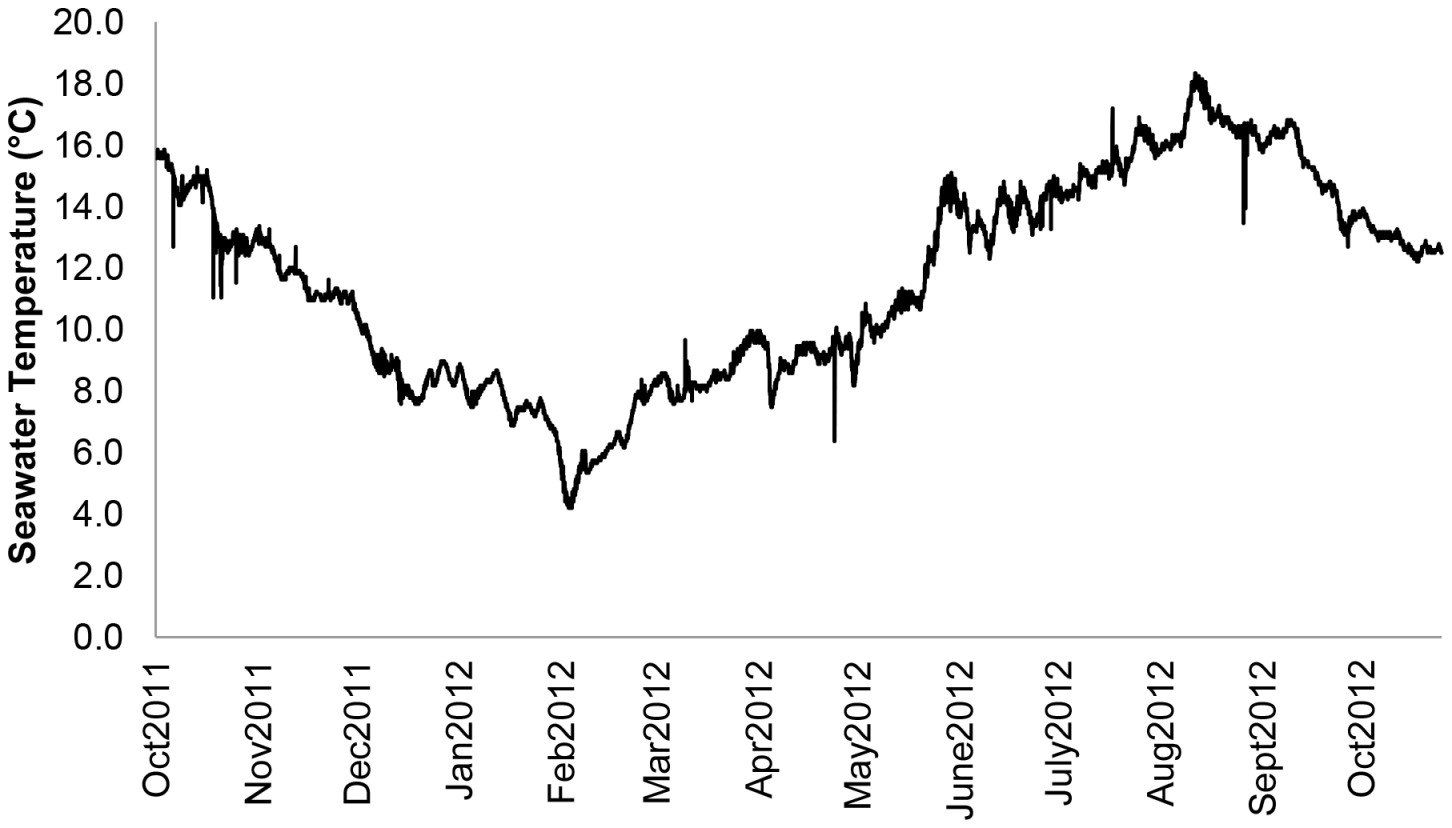
Sub-surface seawater temperature in the Menai Strait (Wales, UK) between October 2011 and October 2012. Temperature was measured at 3

Adult *Mytilus edulis* (L.) were collected subtidally from mussel beds in the Menai Strait, North Wales, UK using a mussel dredge (Deep Dock Ltd.) in May, 2011. Sub-surface (depth 3 m) seawater temperature in the Menai Strait for 2011–2012 as measured with a temperature data logger (Hobo Pendant Temperature Data Logger, Measurement Systems Ltd., Newbury, UK) is presented in Fig. 1. Mussels (640 in total with mean shell length = 50.51±0.15 mm) were transported to aquaria at the School of Ocean Sciences, Bangor University where they were held in aerated seawater in large (200 litre) flow-through holding tanks (12.5±0.26 SD °C, pH 8.01±0.08 SD, Salinity 34±1 SD psu, 12L:12D light regime) for 3 weeks prior to the start of the experiment. The mussels were drip-fed concentrated algal feed (Instant Algae Shellfish Diet 1800, Reed Mariculture, Campbell, CA, USA; 40% *Isochrysis* sp., 15% *Pavlov*a sp., 25% *Tetraselmis* sp. and 20% *Thalassiosir weissglogii*; 52% protein, 16.1% lipid, 22.0% carbohydrate and 9.9% ash) at a ration of 27 mg dry mass mussel^−1^ day^−1^ over a period of 4–6 hours day^−1^.

### Ethics Statement

No specific permits were required for the study, which complied with all relevant regulations. The species collected in this study is not endangered or protected.

### Experimental Set-up

Adult *M. edulis* were exposed for six months in an aquarium-based CO_2_ system to represent present and near-future (i.e. 2100) pH (ambient pH and a decrease of 0.4 pH units) and temperature (ambient 12°C and an increase of 4°C). A factorial experimental design was applied in order to examine the influence of each factor on its own plus their interactive effects. The four treatments were: ambient pH at ambient temperature (ambient); ambient pH at ambient temperature +4°C (warming); reduced pH at ambient temperature (acidified); and reduced pH at ambient temperature +4°C (warming+acidified).

Seawater was pumped from the Menai Strait via an external settling tank to a central indoor sump tank (500 l) held in an air-conditioned (air temperature 12°C) aquarium. Seawater was triple-filtered and UV treated before delivery to each of four header tanks (150 l) representative of the four experimental treatments. The temperature of each header tank was kept constant through use of in-line heaters (Elecro 900 Evo Titanium Digital Aquarium Heater, Elecro Engineering Ltd., Hertfordshire, UK) or flow-through cooling units (Aqua Medic TITAN 2000, Aqua Medic Inc, Loveland, CO, USA). These circulation systems also ensured adequate mixing of the seawater. A reduction in pH was achieved by computer controlled addition of CO_2_ (g). A pH controller (Walchem Dual Input pH Series Controller, Walchem, Holliston, MA, USA) set to end-century pH conditions (pH 7.68) and pH of each high CO_2_ treatment regulated via solenoid valves. A handheld temperature-compensated (HTC) pH meter (Mettler Toledo SG2 SevenGO, MT Ltd., Leicester, UK) was used to measure pH and the pH controller was adjusted accordingly. The HTC pH meter was triple calibrated using Fisher Scientific buffer solutions (pH_NIST_ = 4.01, pH_NIST_ = 7.00, pH_NIST_ = 10.01 at 25°C).

Seawater from each header tank was gravity-fed (flow rate ∼300 ml minute^−1^) to 20 smaller (1.5 l) mussel tanks with overflows running to waste. Eight individual mussels were haphazardly assigned to each tank per treatment. Mussels were acclimated to temperature treatments at an increase of 0.5°C day^−1^. All tanks were cleaned three times per week and mussels were drip-fed concentrated algal feed (Instant Algae Shellfish Diet 1800, Reed Mariculture, Campbell, CA, USA) at a ration of 27 mg dry mass mussel^−1^ day^−1^ over a period of 4–6 hours day^−1^. Mussels were held at a photoperiod of 12L:12D. Shell length, measured to the nearest 0.01 mm with digital callipers (n = 20), was recorded at the beginning and end of the experimental period. Soft tissues were removed from 20 individuals at the start of the experiment to represent baseline values, and then from mussels from each treatment (n = 20) after six months exposure to determine dry tissue mass by drying the total soft tissue at 80°C for 72 h. The condition index (CI) of each mussel was determined as:
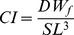
where DW_f_ = mg total soft tissue dry weight, and SL = shell length (cm) after Clausen and Riisgård [32].

### Seawater Parameters

For the analysis of total alkalinity and dissolved inorganic carbon (DIC), seawater samples (60 ml) were taken in triplicate every two weeks in air-tight glass-stoppered containers poisoned with 0.02% mercuric chloride according to procedures outlined by Dumousseaud *et al*. [33]. Temperature and salinity of each sample were also recorded. All samples were analysed by the Carbonate System Facility (LIMS) at National Oceanography Centre, Southampton. Seawater samples (30 ml) were taken at the same time from header mixing tanks, filtered (Whatman GFF 0.7 um) and frozen (−20°C) for the determination of phosphate and silicate concentrations at the Scottish Association for Marine Sciences, Oban, Scotland, UK. Temperature, salinity and carbonate parameters were entered into the CO2SYS model [34] to determine the remaining seawater carbonate parameters (i.e. pH, pCO_2_, HCO_3_, CO_3_^2−^, ΩAr, ΩCa cf Table 1 for explanation) using the thermodynamic constants of Mehrbach *et al.* [35] refitted by Dickson and Millero [36]. Table 1 outlines the carbonate chemistry of each experimental treatment as determined by the CO2SYS model. Additionally, seawater pH and temperature of three randomly selected experimental tanks were measured daily so as to ensure approximate pH and temperature treatments were maintained over the course of the experiment. Prior to seawater pH measurements, the electrode was placed in seawater for approximately one hour to stabilise the liquid junction potential of the electrode [9]. Weekly, nitrate levels were monitored (API Liquid Nitrate Test Kits) and salinity was measured with a refractometer (TMC V2 ATC) calibrated with distilled water prior to use.

**Table 1:** Seawater carbonate chemistry values for the four treatments.

TRT	T (°C)	Sal (‰)	pH_T_	TA(µmol/kg)	DIC(µmol/kg)	pCO_2_(µatm)	HCO_3_^−^(µmol/kg)	CO_3_^−2^(µmol/kg)	Ωarag	Ωcalc
Ambient	12.14 (±0.48)	33.58 (±0.72)	7.99 (±0.05)	2263.57 (±15.70)	2093.78 (±12.69)	465.82 (±63.15)	1959.02 (±30.99)	125.46(±12.67)	1.91 (±0.20)	3.00 (±0.31)
Warming	15.84 (±0.27)	33.71 (±0.69)	7.95 (±0.03)	2263.75 (±15.99)	2087.47 (±13.67)	516.77 (±40.94)	1942.52 (±18.28)	131.45(±8.00)	2.02 (±0.12)	3.15 (±0.19)
Acidified	12.18 (±0.48)	33.63 (±0.71)	7.65 (±0.06)	2264.69 (±16.97)	2213.62 (±19.08)	1087.69 (±169.87)	2113.12 (±21.27)	63.35(±9.57)	0.97 (±0.14)	1.52 (±0.23)
Warming+Acidified	16.11 (±0.28)	33.54 (±0.72)	7.63 (±0.05)	2265.32 (±16.50)	2205.95 (±15.69)	1161.35 (±136.86)	2101.03 (±17.23)	69.07(±7.37)	1.06 (±0.11)	1.66 (±0.17)

Values represent the mean ±SD of bimonthly measures taken over the six months exposure period (n = 12 for each treatment). Mussels were either exposed to: ambient pH and ambient temperature (ambient); ambient pH and elevated temperature (warming); reduced pH and ambient temperature (acidified); or reduced pH and elevated temperature (warming+acidified). TRT represents treatment, T = temperature, Sal = Salinity, pH_T_ = pH (total scale), TA = total alkalinity, DIC = dissolved inorganic carbon, pCO_2_ = CO_2_ partial pressure, HCO_3_^−^ = bicarbonate, CO_3_^2−^ = carbonate, Ω_arag_ = aragonite saturation state, and Ωcalc = calcite saturation state. Measured values are: temperature, salinity, TA, and DIC. Calculated values are pH, pCO_2_, HCO_3_, CO_3_^2−^, Ω_arag_ and Ω_calc_.

### Shell Morphometrics

After six months of exposure, 18 mussels were removed from each treatment group. Following the removal of all flesh, shells were cleaned with de-ionised water and dried at room temperature for 3 weeks. Shell weight was measured with an analytical laboratory balance (Sartorius analytic), and shell length, width, height and thickness (at umbo and distal margin of shell) as defined by Seed [37] were measured with Vernier Callipers (Mitutoyo Series 500, Mitutoyo UK Ltd, Hampshire, UK) to the nearest 0.01 mm.

### Shell Strength

Shell strength was determined on the same shells used for the morphometric measurements (n = 18 per treatment) by measuring the maximum load that each shell valve could endure, and by measuring shell extension properties. Individual load and extension values were calculated as the mean of the two valves for each mussel. Maximum load refers to the highest point on the load-time curve before failure (i.e. fracture) while extension refers to the distance a shell will bend/flex before failure. A decrease in extension may also indicate an increase in brittleness of the shells. Maximum load and extension of individual shell valves were determined using an Instron Universal Testing Machine (Model 2243; 5 kN load cell; 3-point fixture, High Wycombe, Bucks, UK) with loading span of 50 mm and crosshead speed of 5 mm min^−1^ (Illinois Tool Works Inc, IL, USA). All shell valves were placed on the stage of the testing machine in an identical orientation (i.e. shell length along horizontal axis, outer shell surface facing upwards). Maximum loads were recorded at the central point of the outer shell surface. Results were recorded and plotted using Bluehill Software (v.2) (Illinois Tool Works Inc, IL, USA).

### Shell Surface Analysis

A subsample (n = 3 per treatment) of shells from each treatment was subsequently used to determine surface area, as well as to further examine any structural anomalies to the surface of the shells. These additional examinations were carried out to determine whether ocean acidification and/or warming are capable of dissolving the surface of the shells causing surface abrasions and pitting. In order to determine the surface area of a shell valve, mussel shells were dried and then broken into smaller fragments of approximately 5 mm length. To ensure representative results of the whole shell at least three subsamples consisting of a number of shell fragments (of 0.1–0.3 g total mass) per shell were analysed to determine mean values. Analysis was performed on a Geminii Surface Area Analyser (Micromeritecs Instrument Corporation, Georgia, USA) using liquid nitrogen as the coolant. Surface area was determined by nitrogen adsorption on to the surface of the shell fragments. The amount of gas adsorbed at a given pressure allowed for calculation of the surface area utilising the Brunauer, Emmett and Teller (B.E.T.) theory [38].

### Shell Composition

X-ray diffraction was used to determine the mixture of calcium carbonate polymorphs (calcite and aragonite) in each shell. Powder samples of five shells per treatment were analysed using a Bruker AXS D8 Advance X-ray Diffractometer equipped with a superspeed PSD:Vantec-1 detector and CuK_α_ X-ray source. Data was obtained in the 2-theta range 20 to 60°, with a step size of 0.016° and time of 0.538 seconds per step. X-ray diffraction patterns were background corrected using AXS software (Bruker Corporation, Germany). The procedure reported by Kontoyannis and Vagenas [39] was employed for quantitative analysis. This method uses a calibration line constructed from the intensities of the (104) reflection peak for calcite, (C), 2-theta 29.4° and the (221) reflection peak at 2-theta 46° for aragonite, (A). Linear regression of peak intensity, (I), ratios against molar fraction, (X), ratios allowed Kontoyannis and Vagenas [39] to derive the relationship shown in the following equation, which was used to calculate the calcite:aragonite ratios of the shells studied.
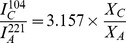
(1)


### Data Analysis

Data was tested for normality and homogeneity of variances (Levene’s Test). If necessary, data was log-transformed to meet assumptions. The significance of the effects of temperature, or the effects of pH, or any interactions between the two factors on the parameters measured were tested by two-way ANOVA. Significant findings were followed by LSD post-hoc tests. All F statistics and p values given in parenthesis in the text represent the results of the respective ANOVAs, and all p values given on their own represent the outcome of subsequent post-hoc tests. Data are presented as means ±SE unless otherwise stated. Statistical analyses were performed using SPSS software (SPSS 14, SPSS INC, Chicago, IL, USA).

## Results

### Condition Index (CI)

**Figure 2:**
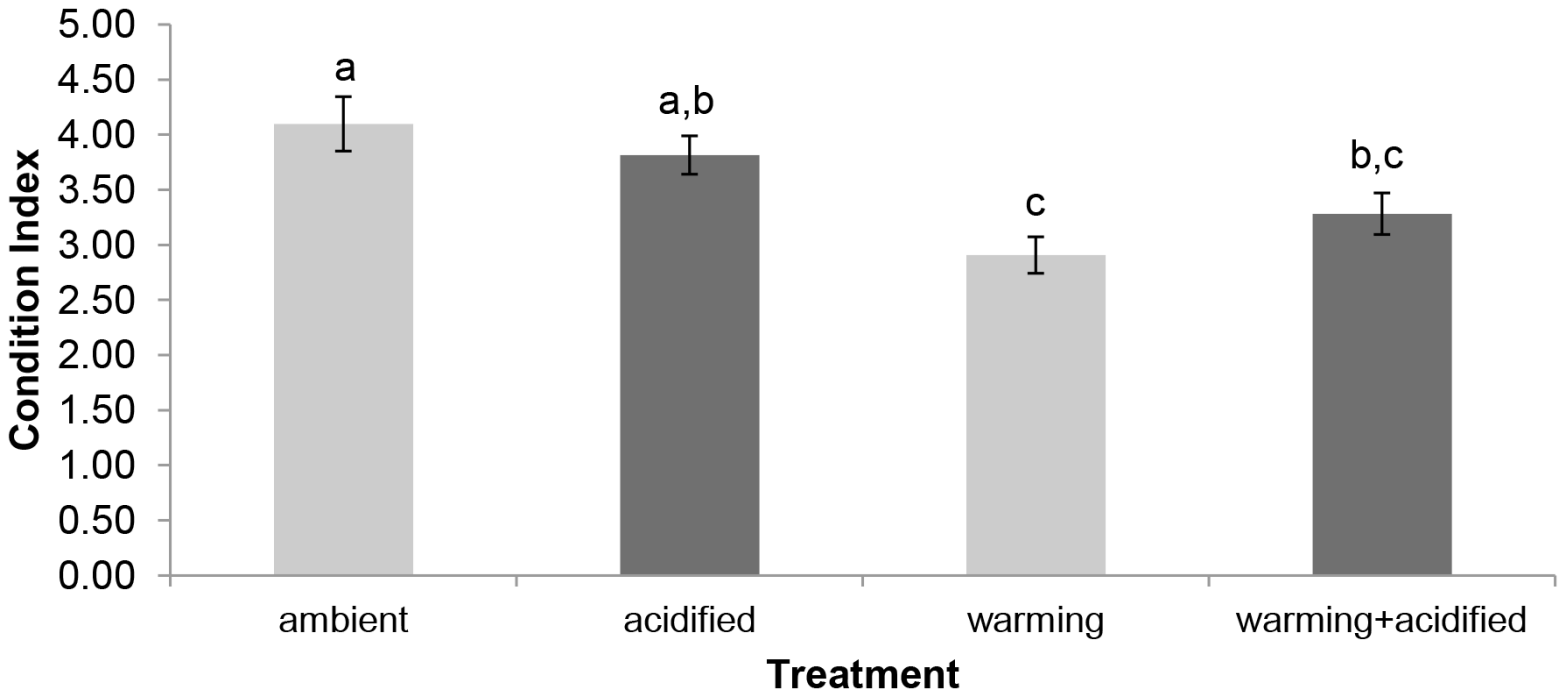
Effects of warming and/or acidification on condition index (CI) in *M. edulis*. Mussels were held for six months under: ambient temperature and ambient pH (ambient); ambient temperature and reduced pH (acidified); elevated temperature and ambient pH (warming); and elevated temperature and reduced pH (warming+acidified).Values given as means **±**SE (n = 20 per treatment). Acidified treatments shown in dark grey. Significant differences indicated by different lowercase letters (p<0.05).

After six months exposure to the treatments, mean CI of adult mussels was significantly affected by temperature (F = 11.869, p = 0.001) but not by pH (F = 0.014, p = 0.906) (Fig. 2). Interaction between the two factors was marginally significant (F = 3.644, p = 0.060). Warming by 4°C resulted in a highly significant reduction in CI from 4.1±0.19 to 2.9±0.17 in mussels held at ambient pH (p<0.001). After six months, CI was significantly lower than the baseline value of 6.44±0.25 (n = 20) for all treatments (p<0.001).

### Shell Morphometrics

After six months exposure temperature had a significant effect on shell thickness (at distal edge), but had no effect on total shell dry weight, length, width, height, and thickness at the umbone (Tables 2 and 3). Mussels held at decreased pH had significantly thicker shells at the distal edge at elevated compared with ambient temperatures (p = 0.008). Seawater pH had no effect of any of the morphological parameters but there was a significant interaction between pH and temperature with regards to shell height (Table 3). Mussels held at ambient pH and elevated temperature (warming) had significantly shorter shell heights than those at elevated temperature and decreased pH (warming+acidified) (p = 0.007) and those at ambient pH and ambient temperature (ambient) (p = 0.018) (Table 2).

**Table 2:** Shell morphometrics and shell composition.

RESPONSE	UNITS	TREATMENT			
		Ambient	Warming	Acidified	Warming+Acidified
**Shell Dry Weight**	g	3.18(±0.17)	2.97(±0.12)	3.07(±0.15)	3.09(±0.16)
**Shell Length**	mm	49.32(±0.85)	49.71 (±0.55)	49.43(±0.82)	50.00(±0.87)
**Shell Width**	mm	23.44(±0.34)	23.55 (±0.18)	23.55(±0.22)	23.95(±0.37)
**Shell Height**	mm	9.30(±0.20)	8.87 (±0.12)	9.17(±0.17)	9.35(±0.22)
**Shell Thickness_U_**	mm	1.40(±0.06)	1.35(±0.06)	1.35(±0.05)	1.35(±0.05)
**Shell Thickness_DE_**	mm	1.10(±0.04)	1.13(±0.05)	1.06(±0.03)	1.17(±0.04)
**Calcite:Aragonite_MF_**	–	2.1(±0.48)	1.7(±0.15)	3.2, 2.9	2.2(±0.39)

Mean values ±SE for shell dry weight, shell length, shell width, shell height and shell thickness (umbo and distal edge), and molar fraction of calcite:aragonite in *M. edulis* following a six month exposure period to four pH-temperature treatments: ambient pH and ambient temperature (ambient); ambient pH and elevated temperature (warming); reduced pH and ambient temperature (acidified); or reduced pH and elevated temperature (warming+acidified). N = 18 per treatment for the morphometric determinations, and N = 4 for shell composition, with the exception of the acidified treatment when only 2 values were recorded and both of these are given. ML: maximum load, E: extension, MF: molar fraction, U: umbo, DE: distal edge.

**Table 3:** Two-way ANOVA results comparing the effect of pH and temperature on shell morphometrics (n = 18).

Source of variation	df	SS	MS	*F*-ratio	*P*-value
**Dry Weight**					
pH	1	0.002	.002	0.006	0.939
Temperature	1	0.361	0.361	0.862	0.355
pH*Temperature	1	0.474	0.474	1.130	0.290
**Length**					
pH	1	1.383	1.383	0.128	0.721
Temperature	1	8.119	8.119	0.753	0.387
pH*Temperature	1	0.281	0.281	0.026	0.872
**Width**					
pH	1	2.341	2.341	1.604	0.207
Temperature	1	2.298	2.298	1.575	0.212
pH*Temperature	1	0.767	0.767	0.525	0.470
**Height**					
pH	1	1.124	1.124	1.970	0.163
Temperature	1	0.515	0.515	0.903	0.344
pH*Temperature	1	3.320	3.320	5.821	0.017*
**Thickness (umbone)**					
pH	1	0.023	0.023	0.449	0.504
Temperature	1	0.022	0.022	0.437	0.510
pH*Temperature	1	0.019	0.019	0.374	0.542
**Thickness (edge)**					
pH	1	0.000	0.000	0.007	0.933
Temperature	1	0.165	6.282	6.282	0.013*
pH*Temperature	1	0.048	1.827	1.827	0.179

Significant differences in bold and presented by an asterisk (p<0.05).

### Shell Strength

**Figure 3:**
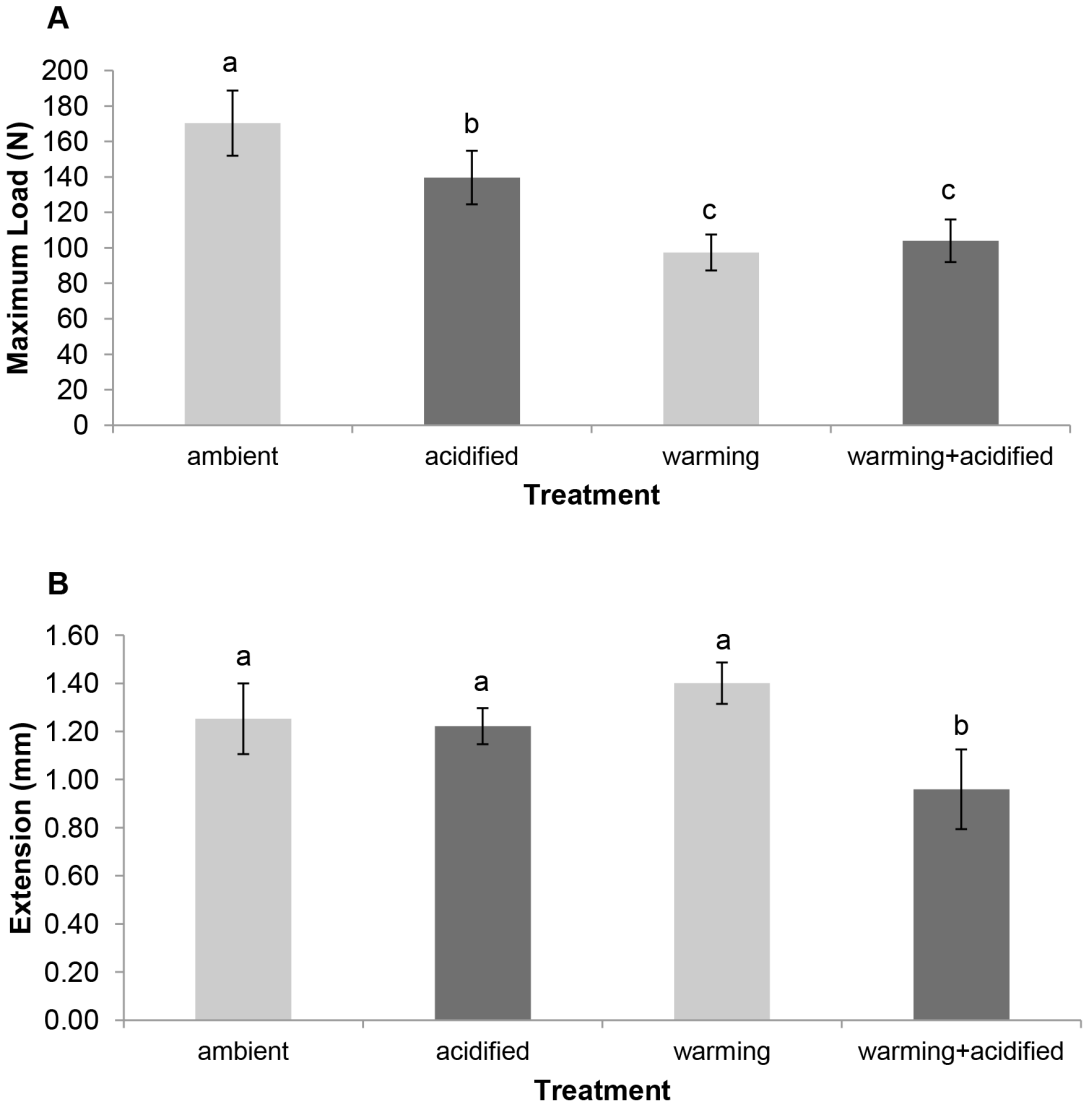
Effects of warming and/or acidification on shell strength. A) Maximum load endured until fracture and B) extension or distance a shell will bend/flex before failure of shells from *M. edulis* held for six months under: ambient temperature and ambient pH (ambient); ambient temperature and reduced pH (acidified); elevated temperature and ambient pH (warming); and elevated temperature and reduced pH (warming+acidified). Values given as means **±**SE (n = 18 per treatment). Acidified treatments shown in dark grey. Significant differences indicated by different lowercase letters (p<0.05).

Two-way ANOVA showed that temperature, but not pH, had a significant effect on the maximum load endured by the mussel shells (F = 31.624, p<0.001 and F = 1.131, p = 0.289, respectively), although there was a near-significant interaction between the two factors (F = 3.590, p = 0.060). Shells from mussels held in ambient conditions (ambient) had significantly higher maximum load values than shells from all other treatments (warming, acidified, warming+acidified) (p<0.001, p = 0.040 and p<0.001, respectively). Likewise, those mussels kept at ambient temperature and reduced pH (acidified) had significantly higher maximum loads than those kept at higher temperature (warming+acidified; warming (p = 0.010 and p = 0.002) (Fig. 3A).

Seawater pH had a significant effect on shell extension values (F = 12.649, p = 0.001), but not temperature (F = 1.113, p = 0.293), and there was a significant interaction between the two factors (F = 6.439, p = 0.012). Shell extension values were significantly lower in mussels held at elevated temperature and decreased pH (warming+acidified) when compared with mussels in all other treatments (ambient, warming, acidified) (p = 0.001, p<0.001 and p = 0.013, respectively) (Fig. 3B).

### Shell Surface Analysis

**Figure 4:**
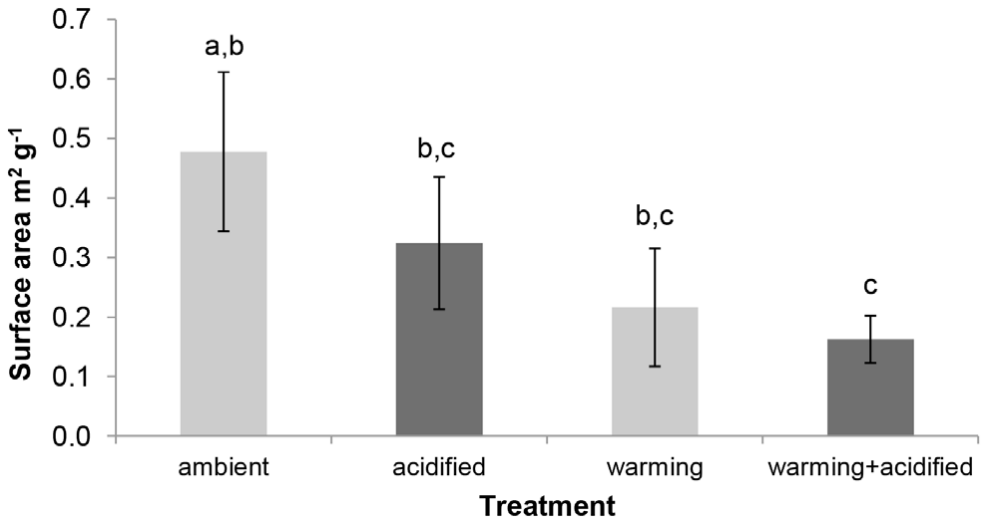
Effects of warming and/or acidification on shell surface area. Shell surface area was determined in at least three subsamples consisting of a number of shell fragments (of 0.1–0.3 g total mass) per shell from *M. edulis* held for six months at either: ambient temperature and ambient pH (ambient); ambient temperature and reduced pH (acidified); elevated temperature and ambient pH (warming); or elevated temperature and reduced pH (warming+acidified). Values given as means **±**SE (n = 3). Acidified treatments shown in dark grey. Significant differences indicated by different lowercase letters (p<0.05).

Changes in surface areas of the shells under the various treatments are given in Fig. 4. Two-way ANOVA showed a significant effect of temperature on shell surface area (F = 10.355, p = 0.003) but neither pH nor the interaction of pH and temperature were significant (F = 2.045, p = 0.162 and F = 0.582, p = 0.451, respectively). The largest mean surface area of the shells at 0.478±0.085 m^2^ g^−1^ was observed in control mussels kept under ambient conditions. The smallest surface areas were obtained in shells from mussels exposed to the combined effects of elevated temperature and low pH (warming+acidified) but these differences were only significant when compared with mussels held at ambient conditions (p = 0.002). At ambient pH, elevated temperature decreased shell surface area by 50%, which was a significant change (p = 0.008).

### Shell Composition

The signal-to-noise ratio on the X-ray diffraction patterns indicated that all samples contained a significant amount of amorphous material, however, it was not possible to quantify this component. Calculations were not carried out for samples where the signal-to-noise ratio was very low, i.e. signal strength was not significantly greater than the background noise. Calcite:aragonite molar fractions are given in Table 2 where n = 4, except for under acidified conditions where only two values were obtained. Two-way ANOVA showed no overall effect of temperature or pH on calcite:aragonite ratios, nor an interaction between the two factors (values for warming+acidified treatment not included).

## Discussion

Data from the maximum loading measurements demonstrated that warming by 4°C for six months had a greater effect on shell strength in *M. edulis* when food was limiting, than a reduction in seawater pH of 0.4 pH units. Consequently, mussels held at 16°C had weaker shells than mussels held at 12°C, regardless of pH level. Shell strength in *M. edulis* has been attributed to material composition and overall shell thickness, size and shape [20]. As the morphology of the shells was generally unaffected by all treatments, the changes in shell strength with temperature observed here cannot be attributed to changes in biometrics. The reduction in shell height during warming at ambient pH was one exception, as was the increase in shell thickness during warming and acidification. Shell height is not normally associated with shell strength, and although shell thickness has been shown to be an effective predictor of strength in *M. edulis* [40], the increase in thickness noted here had no effect on overall strength, and may not be representative of shell thickness in other parts of the shell margin. In addition, preliminary determinations of shell composition using X-ray diffraction of the same mussel shells used for the loading experiments, indicated that the calcite:aragonite molar fraction was unaffected by six months exposure to either warming or acidification. Consequently changes in material composition are unlikely to have been involved, although the possibility that warming affected shell strength via shifts in CaCO_3_ polymorph composition requires more sophisticated investigation. Instead, the present study was able to demonstrate that reductions in shell strength with warming were accompanied by decreases in shell surface area and a fall in whole-body CI.

The reductions in shell surface area with warming recorded here represent surface area reductions of both the outer and inner nacreous layer of the shell fragments. Bivalve shell nacre possesses a deformation mechanism that permits it to exhibit inelastic strain when a force is exerted upon the shell. Inelastic strain allows the nacreous material to redistribute stress around strain concentration sites thereby improving the resistance of the shell to fracture. The level of force at which the inelastic deformation occurs is controlled by mineral asperities on the surface of the aragonite tablets which dictate sliding resistance of neighbouring laminae [41]. In the present study the reduction in shell surface area of the mussels maintained in the warming and acidified condition corresponded with a reduction in shell flexibility. A decreased surface area to volume ratio suggests more extensive shell corrosion and reduced microstructural complexity (e.g. decreased occurrence of asperities, decreased number of laminae, reduced tablet structure) and results in a reduction in the shell’s ability to redistribute stress, thereby reducing material maximum load and extension capabilities.

The maintenance of shell integrity is a dynamic process that is under biological control and takes place in a small compartment, the extrapallial cavity [2], [4]. Shell corrosion is reported to occur on a daily basis in mussels inhabiting the intertidal zone because they are unable to regulate increases in extracellular pCO_2_, which occur at the mantle shell interface during emersion [2], [42]. The resulting extracellular acidosis increases shell dissolution but shell integrity is maintained by continuous shell formation [2]. Shell formation and repair, however, are energetically expensive because of the costs associated with the accumulation, transportation and precipitation of calcium carbonate [43], [44], [45], as well as the costs of the processes involved in the formation of the biologically active, organic matrix [43]. Consequently, shell corrosion in *M. edulis* is related to energy budgets, even under conditions of ambient CO_2_, because shell corrosion is more extensive when food, and therefore energy supply, is limiting both in the laboratory and in the field [2], [46]. In the current study, mussels received 27 mg dry mass mussel^−1^ day^−1^ of algae but were not fed continuously, instead having their entire daily ration delivered over an approximate time span of 4–6 hours. As a result all mussels, even those held in the control treatment of ambient temperature and ambient pH, experienced a decrease in CI which generally indicates a state of lowered “health” [47] and illustrates that the mussels were receiving insufficient food. Moreover, none of the mussels showed any growth over the six month period, regardless of treatment. If restricted access to algae prevented growth and reduced condition in the mussels then exposure to warming could have further increased energy demands at a time when energy supply was limited. Similar temperature-related decreases in bivalve CI have been demonstrated in *M. edulis* and *Arctica islandica* [48], [49], and also in the abalone, *Haliotis iris*, where decreases in shell strength induced by bifacial porosis and endobiont infestations were correlated with reduced CI [50]. The decline in CI observed here suggests that energy reserves were depleted and tissues reabsorbed which typically occurs in bivalves in response to stressful conditions in order to support maintenance energy requirements [51], [52].

Studies on the effects of OA on the growth or calcification rates of shells from *M. edulis* have demonstrated that exposure to the pH levels predicted for 2100 has no effect. Maintenance of shell formation and growth in *M. edulis* despite changes in seawater carbonate chemistry associated with OA has been attributed to a number of factors, such as protection against shell dissolution by the protective organic layer (periostracum), and the mineralogy of the shell [4]. In addition, the ability of *M. edulis* to compensate for the possible dissolution effects of reduced pH on their shells is related to their occupation of habitats characterised by fluctuating pH levels and the capacity of mussels to maintain control rates of shell growth as long as food is in ample supply [46]. The lack of any pH effect on shell morphometrics or shell strength in terms of maximum load capabilities in the present study may also be explained in terms of the cellular model for biomineralisation which maintains that shell formation in molluscs involves the formation of CaCO_3_ crystals in haemocytes which are deposited at the mineralisation front [53], [54]. The involvement of intracellular crystal formation negates the requirement for CaCO_3_ supersaturation in the extracellular compartment where the shell is formed [53], [55], although Roleda *et al*. [55] also argue that HCO_3_^−^ and metabolic CO_2_ are more important to calcification processes than CO_3_^2−^ and hence CaCO_3_ saturation levels. Collectively these studies demonstrate that reductions in seawater pH do not have a direct effect on calcification processes in mussel shells, and this may help to explain the maintenance of shell strength in the present study when mussels were exposed to reduced pH for six months. However, the impact of lowered pH to extension properties of the shell which paralleled temperature effects to shell maximum load suggests that ocean acidification does pose some threat to the condition of the shell. Such decreases may indicate an increased brittleness of the shell brought on by acidified conditions. However the actual mechanisms behind such changes in brittleness require further investigation.

The reduction in shell strength observed here in *M. edulis* is likely to influence survival given the critical role of the shells in prey selection. Predator species have demonstrated an aptitude for testing shell strength and using spot weaknesses to gain access to their food source [56]. Nagarajan *et al.* [21] also demonstrated that predators tend to attack weaker, more thinly-shelled mussels. Therefore, any weakening of shells under future conditions of ocean warming and acidification suggests that *M. edulis* may become increasingly vulnerable to predation. Although *Mytilus* have demonstrated an ability to increase shell thickness and mass, and consequently strength, in high predation sites [57], such compensatory adjustments may not be possible under future warming conditions because of the allocation of energy for temperature-related increases in maintenance requirements. The energy required for compensatory adjustments in shell thickness and mass may be further restricted by any associated declines in food supply that may occur as a result of warming. The observed reductions in soft tissue mass with temperature are concerning because this implies reduced aerobic scope of *M. edulis* and a decline in reserves for ecologically important activities such as growth, fecundity and annual recruitment [31].

In conclusion, it appears that under near-future climate change conditions when food supply is limited, ocean warming will pose a greater risk to shell integrity in the commercial species *M. edulis*, than OA. Whereas food limitation reduced body condition in all mussels, warming by 4°C had a detrimental effect on shell strength which was associated with a reduction in shell surface area. It appears that warming has an indirect effect on shell strength via the re-allocation of energy resources away from costly biomineralisation processes in order to support higher maintenance costs, which may be exacerbated when daily food availability is limited [2], [58]. Seawater acidification had no effect on shell strength but did affect shell flexibility suggesting that shell integrity was mostly maintained in *M. edulis* shells despite reductions in CaCO_3_ saturation. When food is in short supply, *M. edulis* may be at greater risk in its natural environment under future climate change conditions as the shells may not offer sufficient protection of the soft tissues from predators and the rigors of industrial harvesting.
